# Poverty and Pensions

**Published:** 1895-05-11

**Authors:** 


### Modern Sociology.

### POVERTY AND PENSIONS.

The just claim upon the community of the aged and infirm is getting to be more clearly recognised day by day. The public conscience is no longer satisfied that the vast majority of the poor should end their days in a workhouse, and the old dread of making even these too attractive is giving place to some perception of the cruel injustice of embittering the last years of the old folks with conditions only suitable to the penitentiary or the prison.

There is now some danger that small pensions, to be wrung, if possible, eventually out of the State, will come to be regarded as a complete solution of the difficulty, as a kind of panacea, in fact, for all the troubles of old age. Yet, however essential to any scheme for making fit provision for the aged, the pension can only be considered the first step towards their future well-being. Two faults are continually present in the bestowal of the pensions which form an increasing element in the almsgiving of the day. In too many cases a pension of one or two shillings is granted, on proof merely of the poverty and good character of the applicant, without attempt to ascertain how this scanty pittance is to be supplemented. Many cruel instances of long-continued misery, ending at length in death from privation, have resulted from such carelessly-bestowed alms, and, secondly, the recipient is often encouraged to spend on rent a sum out of all reasonable proportion to the total income. It is very usual in the distribution of relief to limit the pension candidates to those living within a certain district and this very probably one where rents are extraordinarily high. Out of twenty-five small and very highly-rented houses within ten minutes of the Marble Arch, there were at one time no fewer than twenty widows, with ages varying from sixty to ninety, and several of them in receipt of 1s. a week pensions which tied them to the neighbourhood. The amount expended on rent by these women for the miserable accommodation they enjoyed, came to about £170 a year. Their combined incomes certainly did not amount to twice as much. In such cases, which are only too common all over London, the pension is simply an encouragement to overcrowd an already congested neighbourhood, and does not in fact suffice to cover the extra rent incurred by enforced residence in the particular district.

The minimum laid down in all old age pension schemes is 5s. a week. How far will this sum go in London? At least 2s. 6d. must be set aside for rent. This will provide a "back-kitchen" (in other words, a small dark cellar), or a "top-back," more airy but impracticable for the aged by reason of stairs and water carrying. The rest will be spent somewhat as follows: Bread and milk a half-penny each a day, or 7d. a week; dinner at 1½d. a day comes to 10½d. a week; ¼ lb. of tea, 4d.; ½ cwt. of coal, 7d.; oil and wood, 1½d. The total is 2s. 6d., and no one will contend that it suffices even for barest necessaries.

But the expense of living in London constitutes only half the evil. Elderly women are peculiarly unsuited for looking after their own interests. They form the prey of their landladies, and too often of their own relatives to an inconceivable extent. They change about restlessly from one room to another, falling often from bad to worse, neglected in illness, and worried beyond expression by the dirt and noise of the tenement house. The monotony of their lives in one little dark room is probably more apparent to the visitor than to themselves, but its loneliness is keenly felt. Their best defence in the varied evil surrounding them is to form no acquaintance and to keep proudly to themselves. Yet the deadly monotony, accompanied as it must be in declining age by increasing infirmity, brings with it inevitable temptation. Close at their door on all sides lie the means of procuring at least momentary solace without risk of detection. And how desperate the temptation, when once yielded to for the occupant of a solitary room, without occupation, and in chronic discomfort, may easily be seen. The mystery rather is, not how many old people succumb and drift down stream after leading respectable lives, but that so many are found sternly to resist and hold up their heads to the last. London is in fact the place of all others where old people should not be, unless provided by their relatives with satisfactory homes. On every ground of their own and the public good they should be induced to remove to purer air and less demoralising surroundings.

Now it is probable that any church or chapel with a weekly list of twenty 1s. pensioners would find that ten out of the number had no actual necessity for remaining in the neighbourhood. For the ten weekly shillings these ten might be comfortably housed in some pleasant village where holders of house property lament in vain the falling off of the population and the impossibility of finding tenants. There would be small difficulty in finding separate accommodation for all in a country house at an outlay of the sum which in London served only to aggravate their poverty, while in almost every village ladies of leisure are to be found willing to befriend such a little colony and help to brighten their lives. Retaining their independence, and mistress each of her little home and individual housekeeping, the old people would enjoy a sense of security and pride of possession impossible to cultivate in the London cellar. The village inn, it may be urged, is still at hand to offer temptation. But there is a wide difference between the secret drinking which in London is made so horribly facile, and the open shame involved by the same act in a little place where every action is known and commented on, and public opinion is stern and wholesome. It is a familiar experience that persons who in London are harassed by a fierce and irresistible craving for drink feel it drop from them in the country like an evil cloud.

As time goes on, and old age pensions in some form or other become an accomplished fact, it is earnestly to be hoped that some such settlements as have been suggested may become a part of the local machinery in every town, parish, and many a country village. No heavy expenditure of capital would be incurred, as in the building and endowment of an almshouse, and a mere handful of subscribers interested in the particular district would suffice to guarantee the rent. The old people need more than money; they need practical assistance in establishing a comfortable environment; they need protection, pure air, quiet, and freedom from anxiety about the future. But they value independence more than all else, and in seeking their true happiness it is above all things important that they shall be left, in all the minor particulars of individual taste, free to order their lives as they will.

